# Shared and distinct peripheral blood immune cell landscape in MCTD, SLE, and pSS

**DOI:** 10.1186/s13578-025-01374-1

**Published:** 2025-04-10

**Authors:** Yanling Cui, Huina Zhang, Yaxuan Deng, Orion Fan, Junbang Wang, Zhonggang Xing, Jianping Tang, Wenmin Zhu, Bangdong Gong, Yi Eve Sun

**Affiliations:** 1 Stem Cell Translational Research Center, Tongji Hospital, School of Medicine, Tongji University, Shanghai, China; 2 Shanghai Institute of Stem Cell Research and Clinical Translation, Shanghai East Hospital, School of Medicine, Tongji University, Shanghai, China; 3 Division of Rheumatology, Tongji Hospital of Tongji University School of Medicine, Shanghai, China

**Keywords:** Mixed connective tissue disease, Systemic lupus erythematosus, Primary Sjögren’s syndrome, Single-cell RNA sequencing, Immune cell landscape

## Abstract

**Background:**

Mixed connective tissue disease (MCTD) is a rare autoimmune disease, and little is known about its pathogenesis. Furthermore, MCTD, systemic lupus erythematosus (SLE), and primary Sjögren’s syndrome (pSS) share many clinical, laboratory, and immunological manifestations. This overlap complicates early diagnosis and accurate treatment.

**Methods:**

The transcriptomic profiling of peripheral blood mononuclear cells (PBMCs) from MCTD patients was performed using both bulk RNA sequencing and single-cell RNA sequencing (scRNA-seq) for the first time. Additionally, we applied MCTD scRNA-seq data, along with datasets from SLE (GSE135779) and pSS (GSE157278) from the Gene Expression Omnibus database, to characterize and compare the similarities and heterogeneity among MCTD, SLE, and pSS.

**Results:**

We first resolved transcriptomic changes in peripheral blood immune cells of MCTD, and then revealed the shared and unique features among MCTD, SLE, and pSS. Analyses showed that the percentage of CD8^+^ effector T cells was increased, while mucosal-associated invariant T cells were decreased in all three diseases. Genes related to the ‘interferon (IFN) γ response’ and ‘IFN α response’ were significantly upregulated. SCENIC analysis revealed activation of STAT1 and IRF7 in disease states, targeting IFN-related genes. The IFN-II signaling network was notably elevated in all three diseases. Unique features of MCTD, SLE, and pSS were also identified.

**Conclusion:**

We dissected the immune landscape of MCTD at single-cell resolution, providing new insights into the development of novel biomarkers and immunotherapies for MCTD. Furthermore, we offer insights into the transcriptomic similarities and heterogeneity across different autoimmune diseases, while highlighting prospective therapeutic targets.

**Supplementary Information:**

The online version contains supplementary material available at 10.1186/s13578-025-01374-1.

## Introduction

Autoimmune diseases (ADs) comprise at least 80 different illnesses, affecting approximately 5% to 8% of the global population. These conditions cause significant suffering for patients and present a major socioeconomic challenge [1, 2]. They all share a common pathogenesis: an immune-mediated attack on the body's own organs. However, there are currently no definitive cures for any of these diseases [3]. Despite numerous efforts to elucidate the underlying pathogenetic mechanisms, the principal causes and effective treatments remain elusive, hampering targeted drug development.

Mixed connective tissue disease (MCTD) is a rare autoimmune disease with a prevalence of 3.8 per 100,000 and a mean annual incidence of 2.1 per million, based on epidemiological data from Norway [4]. It is characterized by overlapping clinical features of systemic lupus erythematosus (SLE), primary Sjögren’s syndrome (pSS), systemic sclerosis (SSc), polymyositis/dermatomyositis (PM/DM), and rheumatoid arthritis (RA), along with high titers of antibodies targeting U1 small nuclear ribonucleoprotein (U1 snRNP) in peripheral blood [5]. Additional distinct clinical features include Raynaud's phenomenon (RP), puffy fingers, polyarthritis, myositis, lung involvement, and esophageal dysmotility. Recent studies have confirmed that MCTD is strongly associated with human leukocyte antigen (HLA), and the central pathogenetic role of anti-U1RNP autoantibodies has clearly emerged [4, 6]. However, due to low prevalence, little is known about the MCTD etiology and pathogenesis [7]. Consequently, there is an urgent need to identify and develop potential therapeutic targets, which requires a deeper understanding of the underlying pathogenesis.

SLE and pSS are among the most common ADs. MCTD, SLE, and pSS share several clinical symptoms, serological profiles, and immunological characteristics [8]. Many MCTD patients may present with the same clinical manifestations as SLE, and SLE may also present with Sjögren’s syndrome (SS) comorbidity [9], including positive anti-SSA and anti-SSB antibodies [10]. These overlapping features hamper disease diagnosis, prognosis estimations, and personalized treatment. Despite advancements in understanding the complexity of disease, such as recognizing its clinical heterogeneity [11, 12], the pathogenesis remains poorly understood, and effective treatments are lacking. Peripheral blood mononuclear cells (PBMCs) include various immune cells that are involved in immune activities and inflammatory responses [13]. Single-cell mRNA sequencing (scRNA-seq) offers a powerful and unbiased approach to profile the cellular composition and specific transcriptional states of PBMCs [14, 15]. Delving into the shared and distinct peripheral immune cell atlas could further clarify the pathogenetic mechanisms and provide a biological basis for treating MCTD, SLE, and pSS.

This study provides a panoramic PBMCs map by combining bulk RNA sequencing (bulk RNA-seq) and scRNA-seq analyses, revealing changes in cell type proportions, gene profiles, biological features, transcription factor (TF) activity, and cellular interactions involved in MCTD. The transcriptomics data presented here elucidated mechanistic features and provided some insights into MCTD pathogenesis. To further understand the pathogenesis of MCTD, SLE, and pSS, several existing human scRNA-seq datasets were compared to construct a comprehensive PBMC transcriptome. Both shared and distinct cell proportions and transcriptional changes among MCTD, SLE, and pSS were revealed. The proportions of CD8^+^ effector T cells were increased, while mucosal-associated invariant T (MAIT) cells were decreased in all three diseases. Upregulated interferon α (IFN α) and IFN γ responses were identified as shared transcriptomic characteristics across MCTD, SLE, and pSS. STAT1 and IRF7 might be shared core TFs involved in regulating the IFN responses. The IFN-II signaling network was found to be highly enriched in MCTD, SLE and pSS. In addition, analyses also revealed the unique features in MCTD, SLE, and pSS.

Altogether, these analyses help to characterize common mechanisms in the immunopathogenesis of MCTD, SLE, and pSS, and identify new potential therapeutic targets.

## Materials and methods

### Subjects

Patients were all recruited from the Department of Rheumatology and Immunology, Shanghai Tongji Hospital. The inclusion criteria for MCTD patients were (1) age ≥ 18 years at the time of diagnosis; (2) a clinical diagnosis of MCTD verified by a rheumatologist; (3) a positive anti-U1RNP test; and (4) fulfillment of at least one of the three criteria sets for MCTD [4] (modified Sharps criteria, Alarcón-Segovia criteria or Kasukawa criteria). All the enrolled MCTD patients were treatment-naïve and had not receive glucocorticoid (GC) or immunosuppressant treatment before blood collection. Exclusion criteria included active infection, a history of malignancy, lymphoma, or hematological diseases, the presence of other autoimmune diseases, pregnancy or lactation in females, and patients whom physicians recommended should not be included. The demographics and baseline disease characteristics are described in Supplementary Tables 1 and 2. SLE patients met the 2019 American College of Rheumatology/European League Against Rheumatism (ACR/EULAR) classification criteria for SLE [16]. All pSS patients met the 2016 ACR/EULAR classification criteria [17]. The demographic characteristics, baseline clinical characteristics, and previous treatment history of the patients are shown in Supplementary Tables 3 and 4. Patients aged 18 to 65 years were enrolled, and healthy donors within the same age range were recruited as healthy controls (HCs). All subjects underwent a physical examination and completed a questionnaire by trained doctors. This study was approved by the Ethics Committee of Shanghai Tongji Hospital (2019Hdx173). Written informed consents were obtained from all participants before enrollment.

### Human blood acquisition

MCTD patients, SLE patients, pSS patients, and HCs were recruited in this study. Blood samples were obtained from patients and HCs and used for scRNA-seq or qPCR analysis. PBMCs were isolated using density gradient centrifugation with Ficoll-Paque ™ PLUS Media. The PBMCs were washed, counted and stored for subsequent experiments.

### RNA isolation and quantitative real-time reverse transcription PCR (qRT-PCR)

Blood samples were obtained from HCs, MCTD patients, SLE patients, and pSS patients. Total RNA was extracted from peripheral blood using TRIzol LS (Invitrogen) according to the manufacturer’s instructions. RNA quality was assessed using NanoDrop spectrophotometer and an Agilent 2100 Bioanalyzer (Thermo Fisher Scientific). RNA was reverse transcribed into cDNA using a reverse transcription kit (Vazyme, R323) according to the manufacturer’s specifications. Real-time PCR was performed using 2 × Taq Pro Universal SYBR qPCR Master Mix (Vazyme, Q712) in ABI Prism 7300 Thermal Cycler (Applied Biosystems) on cDNA sample. Relative expression was determined by calculating the formula 2^−ΔΔCt^. The primers used for gene expression are listed in Supplementary Table 5.

### Bulk RNA sequencing (Bulk RNA-seq)

Sequencing libraries were generated using the NEBNext^®^ UltraTM RNA Library Prep Kit for Illumina^®^ (NEB, USA) following the manufacturer’s recommendations. The libraries were sequenced on the Novaseq 6000 platform (Illumina), generating 150 bp paired-end reads. Raw data in FASTQ format were first processed. After removing adapters, ploy-N tails, and low-quality reads, clean data were obtained and aligned to the human reference genome (GRCh38). We used the HISAT2-StringTie-featureCounts-pipeline to process data [18, 19]. Gene expression levels were estimated by summarizing counts at the gene level.

### Differential gene expression and functional enrichment analyses

Differential gene expression analysis was performed using the DESeq2 R package. *P*-values were adjusted using Benjamini and Hochberg’s approach to control the false discovery rate (FDR). Genes with adjusted *p*-values (FDR) < 0.05 and log2FC (fold change) ≥ 0.5 were considered differentially expressed. Volcano plots were created using the ggplot2 R package to visualize the identified differentially expression genes (DEGs). Hallmark gene sets represented biological states or processes derived from the Molecular Signatures Database (MSigDB) [20]. The “clusterProfiler” R package was used to conduct Gene ontology (GO) and Hallmark functional annotation analyses. Significantly enriched outcomes were identified based on *p*-values < 0.05.

### Single cell RNA sequencing (scRNA-seq)

PBMCs were quickly thawed at 37 °C and resuspended in RPMI-1640 supplemented with 10% FBS. Cells viability was determined using trypan blue staining, and samples with viable rates less than 70% were excluded. Single-cell capture and library construction were performed using the Chromium Single Cell 5 ′ Library & Gel Bead kit (10 × Genomics) according to the manufacturer’s protocols. The cDNA libraries were sequenced using the Illumina NovaSeq 6000 platform. Raw sequencing data were processed using the CellRanger pipeline [21] and aligned to the human reference genome (GrCh38) to generate a raw unique molecular identifier (UMI) count matrix, which was converted into a Seurat object using the Seurat [22] R package.

### scRNA-seq data analysis

The preliminary cell-gene matrix was used for downstream analyses. The pipeline utilized to process the data was performed as follows: Briefly, cells with fewer than 200 genes and more than 10% mitochondria-related genes were filtered out. Logarithmic normalization of counts and selection of the top 3000 highly variable genes (HVGs) were performed using Seurat. R package scDblFinder was used to remove doublets, and the Harmony algorithm was applied to correct for batch effects. Principal component analysis (PCA) was conducted for dimension reduction, and the elbow plot function was used to determine the number of principal components (PCs) to use for clustering on an integrated data matrix. Cells were then clustered using the FindNeighbors and FindClusters functions, and the resulting clusters were visualized using a uniform manifold approximation and projection (UMAP) plot. Cell types were identified using classical marker genes and the SingleR algorithm [23], referring to the Monaco immune datasets.

### Comparing immune cells proportion

For PBMCs, the proportion of immune cells for each cell type was calculated. The proportion for each sample was determined by dividing the number of cells of a particular cell type by the total number of cells. To identify changes in cell proportions between samples from different groups, we performed Mann–Whitney U test on the proportions of each cell type across the groups. Only cell types with statistically significant differences (*p* < 0.05) in proportions were shown.

### Differential gene expression, over-representation analysis and score signature modules

To identify the DEGs across different cell types from PBMCs in patients with MCTD, SLE, and pSS, the FindMarker function (Logfc.threshold = 0.25, *p* < 0.05) in Seurat was used. Over-representation analysis of DEGs (logFC > 0.5 and adjusted *p* < 0.05) was performed using “clusterProfiler” with “HALLMARK” gene sets derived from MSigDB [20]. *p* < 0.05 was considered significant. Signature module scores were calculated using the “AddModuleScore” function with default settings in Seurat. Briefly, for each cell, the score was defined as the average expression of the signature gene list minus the average expression of the corresponding control gene list [24]. The *p*-value for the comparison between two groups was calculated using the Wilcoxon rank test. Gene lists used for analysis were referenced from the MSigDB database.

### Transcriptional regulation analysis

Single-cell regulatory network inference and clustering (SCENIC) was used to predict TFs and their corresponding target genes, construct TF regulatory network modules (regulons), quantify the activity of each regulon in cells based on the expression of TFs and target genes in the regulon, and ultimately determine the transcriptional activity of TFs in different cells. SCENIC was executed from the raw counts and followed the recommended workflow by using default parameters [25]. The analysis involved a three-step process: GRNBoost, RcisTarget, and AUCell. For visualization, the scores of average regulon activity (AUC) for each cell type were calculated, and a rank plot of regulons was drawn using ggplot2. Regulon specificity scores (RSS) were calculated by the “calcRSS” function from the SCENIC algorithm with default parameters. The network of regulons and their target genes was constructed using Cytoscape [26].

### Cell–cell interaction analysis

CellChat was performed to identify and visualize cell–cell interactions among distinct immune cells under different conditions. The official workflow was followed to load the normalized counts from Seurat into CellChat and applied the standard preprocessing steps [27]. After creating CellChat objects, CellChatDB.human was used as the database. Default parameters were then used to identify putative interaction pairs, and the results were displayed as circos plots. Cellular communications across different cell types were identified based on the gene expression of ligands in one cell subpopulation and expression of specific receptors in another cell subpopulation.

### scRNA-seq comparative analysis among MCTD, SLE and pSS

We analyzed the scRNA-seq dataset GSE157278 for PBMCs from 5 pSS patients and 5 HCs. Correspondingly, a matching scRNA-seq dataset GSE135779 for PBMCs from 5 adult SLE patients and 5 adult HCs was downloaded for further comparisons. The same pipeline was used to process both datasets. In brief, quality control was performed by filtering cells with nFeature_RNA > 200 and percent.mt < 10. After normalization, the analysis was conducted on the top 3000 HVGs in each sample following variance-stabilizing transformation. Data integration was carried out using the Harmony function, scaling was done with the ScaleData function, and dimension reduction was performed with the RunPCA function. Finally, cell clustering was achieved using the FindNeighbors and FindClusters functions.

### Availability of data and materials

Public data used in this work are available from the NCBI Gene Expression Omnibus (GEO) under the Accession number GSE135779 and GSE157278. Other data supporting the findings of this study are available from the corresponding author upon reasonable request.

### Statistics analysis

All statistical analyses were performed using Prism (GraphPad, v.8.2.1) and R software (v.4.1.0). Statistical significance was assessed using the Mann–Whitney U test. Differences were considered significant when *p < 0.05, **p < 0.01, ***p < 0.001 and ****p < 0.0001.

## Results

### scRNA-seq revealed altered PBMC composition in MCTD patients

**Fig. 1:**
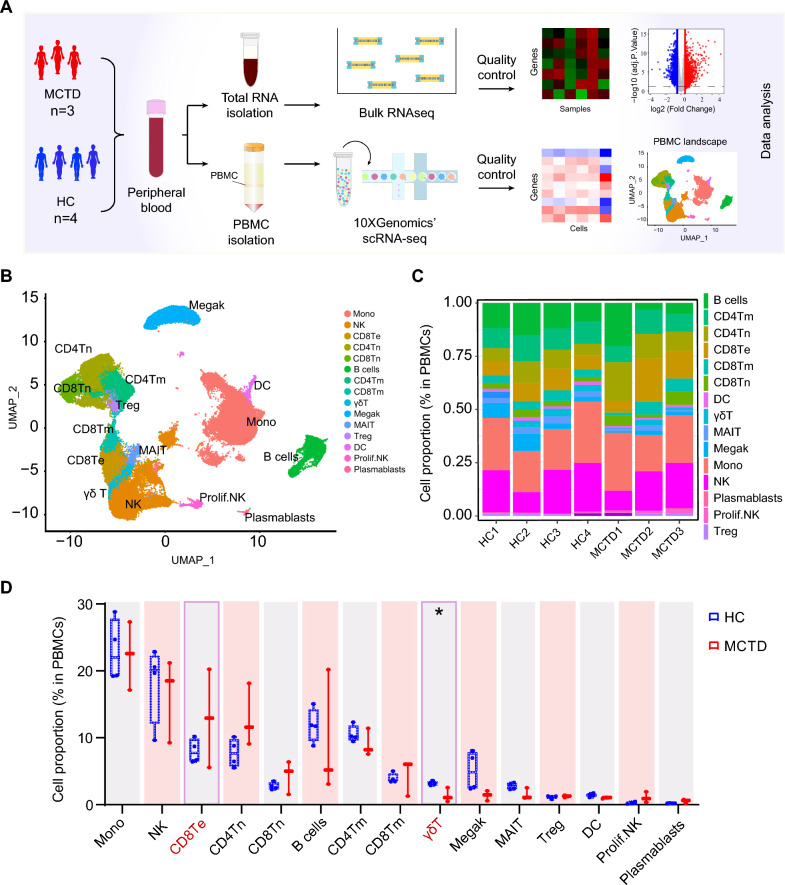
Study design and single-cell transcriptomic profiling of PBMCs from MCTD and HCs. **A** Experimental design and data processing workflow. **B** The uniform manifold approximation and projection (UMAP) plot of single-cell profile for MCTD. Each cell type is distinguished by a different color. **C** Bar plot showing the proportions of cell types in each sample. **D** Box and whisker plot showing the fraction of cell types in MCTD and HCs. *p < 0.05

To explore the immune responses in peripheral blood, blood samples from three MCTD patients and four HCs were obtained, and PBMCs were sorted and subjected to scRNA-seq using the 10X Genomics Chromium platform (Fig. 1A). After quality control and removal of hybrid transcriptomes (multiplets), the HCs PBMCs and MCTD PBMCs were combined and then corrected for batch effect. An atlas was constructed comprising 41,424 cells. Cluster analysis identified 15 distinct cell types. Based on the expression of classical marker genes and the SingleR algorithm, these clusters were designated as monocytes, B cells, NK cells, mucosal-associated invariant T (MAIT) cells, T regulatory cells (Treg), Gamma-delta (γδ) T cells, dendritic cells (DCs), megakaryocytes (Megak), CD4^+^ naïve T cells (CD4^+^ Tn), CD4^+^ memory T cells (CD4^+^ Tm), CD8^+^ naïve T cells (CD8^+^ Tn), CD8^+^ memory T cells (CD8^+^ Tm), CD8^+^ effector T cells (CD8^+^ Te), proliferating NK cells (prolif. NK) and plasmablasts (Supplementary 1, Fig. 1B). The cell proportions in each sample were shown in Fig. 1C. The percentage of each cell type in each group is shown in Fig. 1D. A decrease in γδ T cells (*p* < 0.05) and an increase in CD8^+^ Te cells in MCTD patients were observed, although the latter did not reach statistical significance. The remaining subsets were present in similar proportions across the two groups. Thus, scRNA-seq analysis revealed cell heterogeneity in MCTD.

### MCTD patients were characterized by an elevated response to IFN

**Fig. 2:**
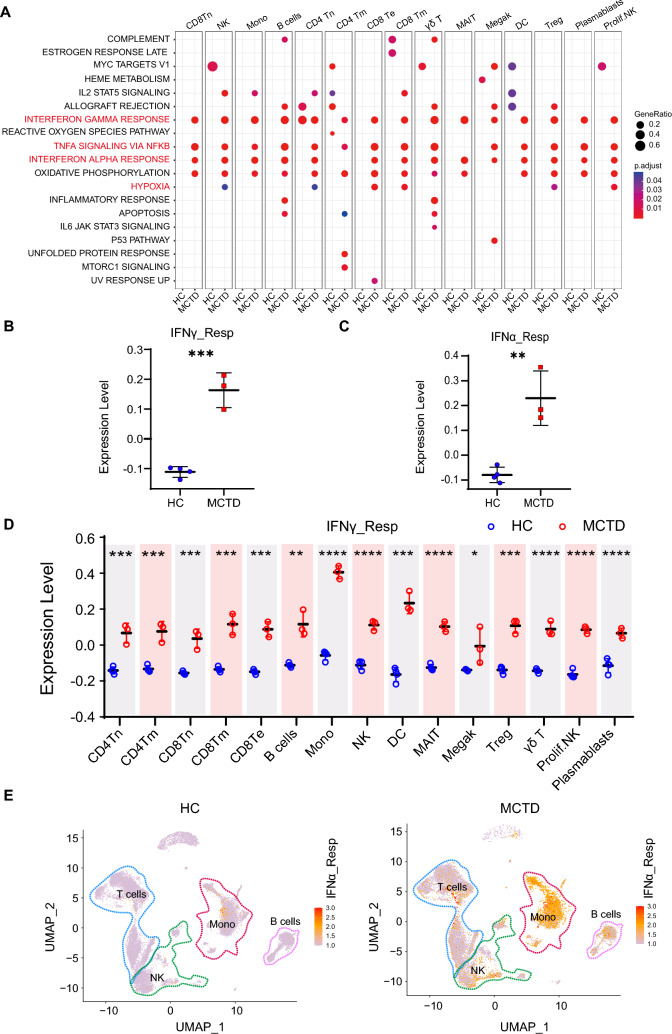
Differential gene expression and over-representation analysis between MCTD and HCs. **A** Over-representation analysis of HALLMARK gene sets for each cell type’s DEGs in MCTD and HCs. Scatter dot plot depicting **B** “IFN γ response” expression score and **C** “IFN α response” expression score between MCTD and HCs. **D** Scatter dot plot depicting the “IFN γ response” expression score of each cell type between MCTD and HCs. **E** UMAP visualization colored by the “IFN α response” expression score between MCTD and HCs

DEGs across all cell types in scRNA-seq between MCTD patients and HCs were identified, and gene functional analysis was conducted. Significantly upregulated genes in MCTD were associated with “IFN γ response”, “IFNα response”, “TNFα signaling via NF-κB”, and “hypoxia” (Fig. 2A). DEG analysis between MCTD and HCs was also performed using RNA-seq data. The volcano plot described the identified DEGs, and the bar plot showed the GO analysis results (Supplementary Fig. 2A, B). Echoing the scRNA-seq analysis results, genes related to “IFN γ response”, “IFN α response”, “TNFα signaling via NF-κB”, and “hypoxia” were upregulated. This finding strongly indicated that IFN response and cytokine stimulus participated in MCTD development and progression. To visualize which pathways were significantly enriched in MCTD, gene module scores were calculated and displayed using scatter dot plot. Interestingly, genes related to “IFN γ response” and “IFN α response” were significantly highly expressed in MCTD patients (Fig. 2B, C). The cell types significantly enriched for these pathways were further visualized by displaying the scores on UMAP coordinates, as well as in grouped scatter dot plots for each cell type (Fig. 2D, E and Supplementary Fig. 2C, D). Notably, genes related to the 'IFN γ response' and 'IFN α response' were significantly highly expressed in monocytes from MCTD patients, suggesting that monocytes are one of the major cell types participating in IFN responses in MCTD. These results were consistent with previous study [28]. Meanwhile, genes related to “TNFα signaling via NF-κB” and “hypoxia” showed an upregulated trend without significant differences (Supplementary Fig. 3).

### TF STAT1 and IRF7 could promote the IFN response

**Fig. 3:**
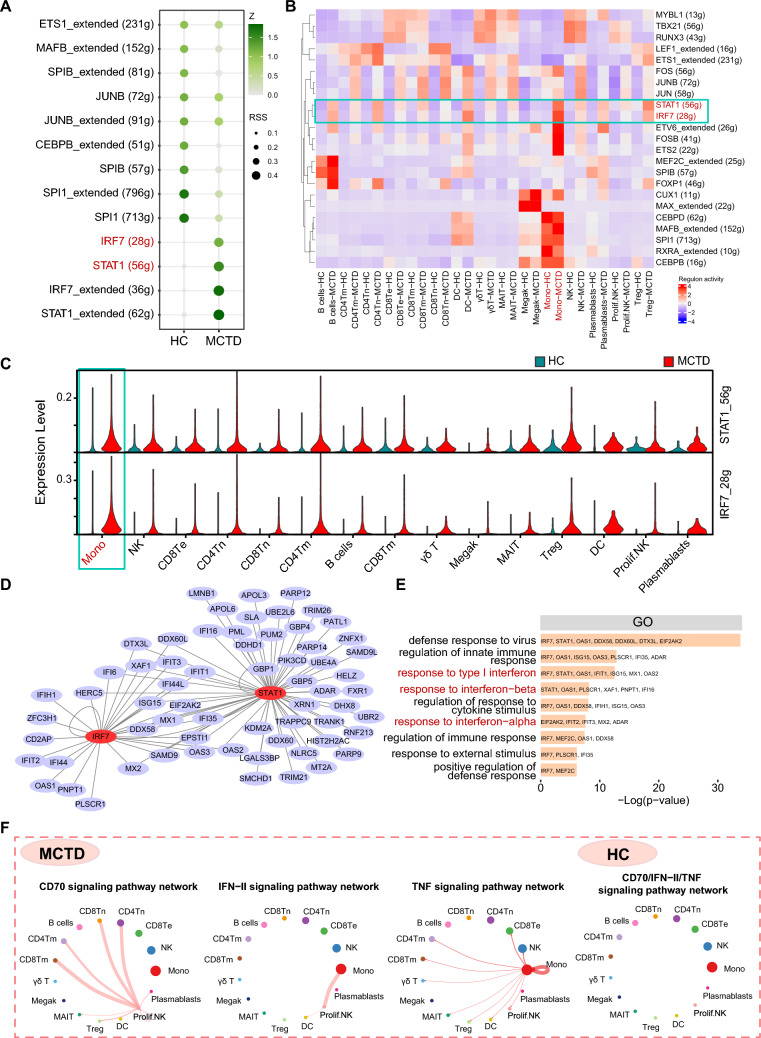
The gene regulatory networks and intercellular communications between MCTD and HCs. **A** The regulon specificity score (RSS) of regulons between MCTD and HCs. **B** The AUCell scored the activity of regulons in each cell type between MCTD and HCs. **C** Violin plot of TF motif expression levels in each cell type between MCTD and HCs. **D** Network of regulons and their target genes. Red indicates TFs and purple indicates target genes. **E** Gene functional annotation of the TF target genes. **F** Circle plots showing the uniquely increased signaling pathway network in MCTD

Given that the gene expression was significantly altered in all cell types, it was hypothesized that there might be some TFs acting as master regulators leading to immunological alterations. SCENIC was used to map the gene regulatory networks and identify potential TF. Group-specific regulons were analyzed using the regulon specificity score (RSS) and regulon activity was assessed with AUCell in each cell type (Fig. 3A, B). The activities of STAT1 and IRF7 were increased in MCTD across all cell types, particularly in monocytes. The expression levels of STAT1 and IRF7 motifs were also elevated in each cell type (Fig. 3C). Furthermore, regulatory network analyses were conducted to identify target genes (Fig. 3D). The GO terms for the target genes were predominantly related to IFN response, including “response to type I IFN” and “response to IFN α/β” (Fig. 3E). This suggested that IFN responses were upregulated in the peripheral immune system in MCTD patients, driven by increased activities of the regulons STAT1 and IRF7, which are the putative master TFs for type I and III IFN signaling [29].

### Communication among immune cells

Excluding cell-intrinsic information, scRNA-seq can also indicate putative cell-extrinsic interactions by integrating ligand and receptor information. CellChat was utilized to investigate the putative interactions between immune cells in MCTD versus HCs. Global cell-to-cell communication networks among the 15 immune cell types were established by counting the number and strength of interactions based on ligand-receptor pairs in MCTD and HCs, respectively (Supplementary 4B, 4C), which were increased in the MCTD group (Supplementary 4A). Many significant ligand–receptor pairs were detected among the 15 cell types. Circos plots were used to visualize specific interactions among the 15-cell group. The results showed increased activity in pathways, including CD70, IFN-II, and TNF, uniquely in MCTD patients (Fig. 3F). Furthermore, monocytes were prominently influenced by the IFN-II signaling pathway (primarily IFN γ-IFN γ R1/R2). The CD4^+^ Tn, CD4^+^ Tm, CD8^+^ Tn, and CD8^+^ Tm cells exhibited the properties of target cells (receiver), while the prolif. NK cells acted as source cells (sender) in the CD70 signaling pathway. Monocytes were presented the dominant source cells (sender) of the TNF signaling pathway. These findings were consistent with the previously observed biological features in MCTD.

### Immune cell heterogeneity in MCTD, SLE, and pSS

**Fig. 4:**
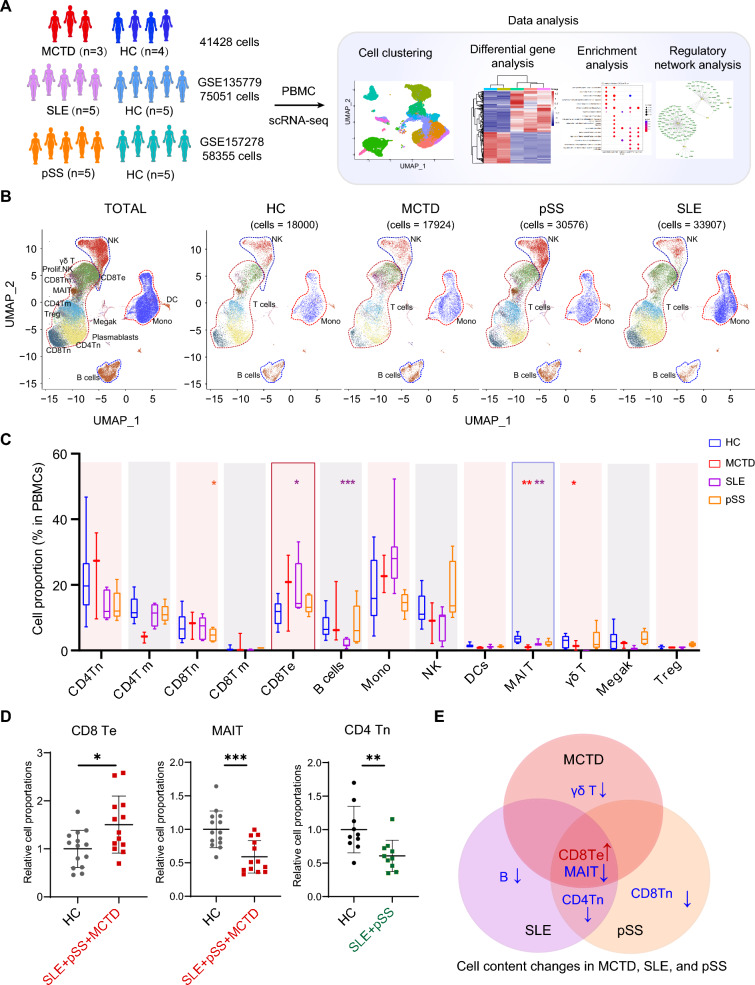
Single-cell transcriptional landscape of MCTD, SLE, and pSS. **A** Experimental design and workflow of the study. **B** UMAP embedding of the entire dataset colored by generated clusters, labelled by cell type annotation and split by each AD as well as HCs. **C** Box plot showing the fraction of cell types in MCTD, SLE, and pSS. **D** Percentages of specific immune cell subtypes in total PBMCs from each individual. *p* values were calculated using the Mann–Whitney U test for comparisons between HCs and disease groups. *p < 0.05, **p < 0.01, ***p < 0.001. **E** Venn diagram of shared and distinct changes in cell proportions among MCTD, SLE, and pSS

To understand immune cell heterogeneity in patients with ADs, scRNA-seq datasets for PBMCs from SLE and pSS patients were downloaded from the GEO database for further comparisons. Since the GSE157278 dataset included 5 pSS patients and 5 normal controls, 5 HCs and 5 SLE patients were selected from the GSE135779 dataset (Fig. 4A). The datasets were divided into subset (18,000 cells in HCs, set.seed = 3) and an integrated analysis was performed to merge the HCs data with the MCTD, SLE, and pSS datasets using the Seurat package integration pipeline (Fig. 4B). Cell type proportions were estimated across disease groups. CD8^+^ Tn cells were significantly reduced in pSS patients, while a significant decrease in B cells and an increase in CD8^+^ Te cells were observed in SLE patients. MAIT cells were significantly decreased in both MCTD and SLE patients, whereas γδ T cells were significantly reduced in MCTD patients compared to HCs (Fig. 4C). Interestingly, CD8^+^ Te cells were expanded in MCTD and pSS patients, while MAIT cells were decreased in pSS patients relative to HCs. Additionally, CD4^+^ Tn cells were reduced in both SLE and pSS patients (Fig. 4C). However, possibly due to the small sample size, these trends were not statistically significant. Therefore, after normalizing the data, we combined the disease groups and examined the changes in cell proportions (Fig. 4D). CD8^+^ Te cells were markedly expanded, while MAIT cells were decreased in MCTD, SLE, and pSS patients relative to HCs. Additionally, CD4^+^ Tn cells were also reduced in SLE and pSS patients. In summary, MCTD, SLE, and pSS shared common changes in immune cell composition, including an increased proportion of CD8^+^ Te cells and a decreased proportion of MAIT cells. A reduction in CD4^+^ Tn cells was a common change observed in both SLE and pSS. Meanwhile, the decreased proportion of γδ T cells, B cells, and CD8^+^ Tn cells was distinctly characteristic of MCTD, SLE, and pSS, respectively (Fig. 4E).

### Identification of shared and distinct biological features in MCTD, SLE, and pSS

**Fig. 5:**
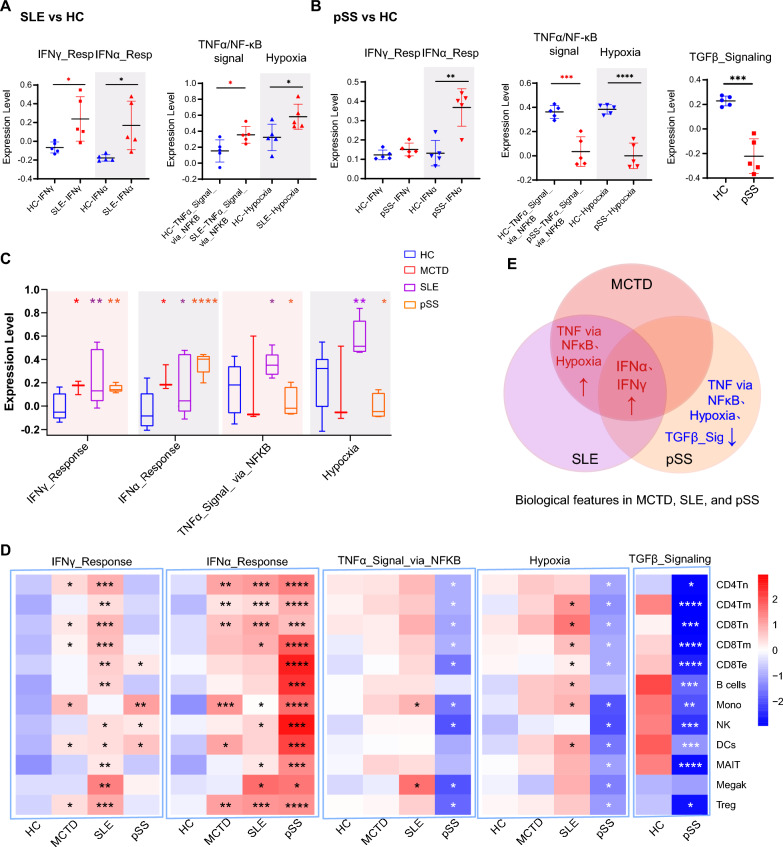
The average expression scores of specific enrichment pathways. **A** Scatter dot plot depicting “IFN γ response”, “IFN α response”, “TNFα signaling via NF-κB”, and “Hypoxia” expression scores between SLE and HCs. **B** Scatter dot plot depicting “IFN γ response”, “IFN α response”, “TNFα signaling via NF-κB”, “Hypoxia”, and “TGFβ signaling” expression score between pSS and HCs. **C** Bar plot depicting “IFN γ response”, “IFN α response”, “TNFα signaling via NF-κB”, and “Hypoxia” expression scores across MCTD, SLE, pSS, and HCs. **D** Heatmap showing the expression score of “IFN γ response”, “IFN α response”, “TNFα signaling via NF-κB”, and “Hypoxia” in each cell type across MCTD, SLE, pSS, and HCs. **E** Venn diagram showing the shared and distinct changes in biological features among the three diseases

To understand the shared and distinct biological features in patients with ADs, DEGs for each immune cell type were screened between the SLE group and HCs group, as well as between the pSS group and the HCs group, and gene functional analyses were conducted accordingly. Genes related to “TNFα signaling via NF-κB”, “IFN γ response”, “IFN α response”, and “hypoxia” were upregulated in the SLE group compared to the HCs group (Supplementary Fig. 5A). Compared to the HCs group, genes related to “IFN γ response” and “IFN α response” in the pSS group were increased, while genes related to “TNFα signaling via NF-κB”, “hypoxia”, and “TGFβ signaling” pathways were decreased (Supplementary Fig. 5B). To evaluate the significance and visualize which cell types were enriched for these signatures, gene module scoring was performed in the groups and the scores were displayed using heatmaps and scatter dot plots. Genes related to “IFN γ response”, “IFN α response”, “TNFα signaling via NF-κB” and “hypoxia” (Fig. 5A, Supplementary Fig. 6A, B) were highly expressed in the SLE patients. In pSS patients, genes related to “IFN γ response” and “IFN α response” were upregulated (Fig. 5B, Supplementary Fig. 6C). In contrast, genes related to “TNFα signaling via NF-κB” and “hypoxia” were downregulated in the pSS group (Fig. 5B, Supplementary 6D). Additionally, “TGFβ signaling” related genes were also downregulated (Fig. 5B, Supplementary 6E). Collectively, genes related to “IFN γ response” and “IFN α response” were commonly upregulated in MCTD, SLE, and pSS. These results confirm the vital roles of IFN in the pathogenesis of AD [28, 30–34]. Genes related to “TNFα signaling via NF-κB” and “hypoxia” were significantly increased or decreased in SLE or pSS, respectively, while showing an upregulated trend in MCTD (Fig. 5C, D). Additionally, the downregulation of “TGFβ signaling” related genes was a characteristic of pSS [35, 36] (Fig. 5D, E).

### Common TFs were responsive to the biological features in MCTD, SLE, and pSS

**Fig. 6:**
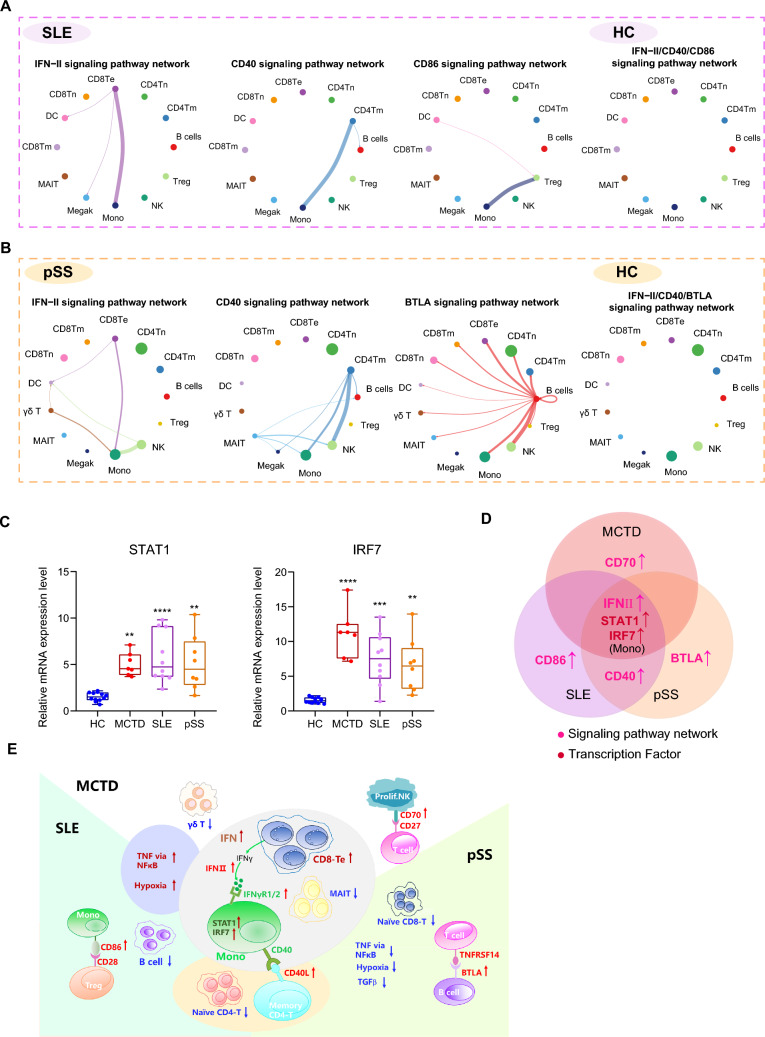
The comparison of TFs and signaling pathways among MCTD, SLE, and pSS. Circle plots showing the uniquely increased signaling pathway network in **A** SLE and **B** pSS. **C** The relative mRNA expression levels of STAT1 and IRF7 in MCTD, SLE, and pSS. **D** Venn diagram showing the shared and distinct cellular interaction signaling pathways and TFs among the three diseases. Magenta indicates cellular interaction signaling, and red indicates TFs. **E** Schematic diagram illustrating the shared and distinct changes in peripheral blood immune status characteristics among MCTD, SLE, and pSS

To elucidate TFs acting on gene enrichment in ADs, SCENIC was used to map the gene regulatory networks governing different diseases and to identify potential TFs modulating DEGs in these diseases. Regulon activities were scored using AUCell to assess the average enrichment of all genes belonging to each regulon in each cell type, as well as across different groups. Shared regulons in SLE and pSS diseases were identified by AUCell score as candidate TFs underlying the gene expression differences in different cells, as discussed in our previous article [37]. The activities of STAT1 and IRF7 were upregulated mainly in monocyte from both SLE and pSS patients [37]. RT-qPCR was further employed to measure the relative mRNA expression levels of STAT1 and IRF7. Both STAT1 and IRF7 were significantly upregulated in MCTD, SLE, and pSS (Fig. 6C). Overall, the upregulation of STAT1 and IRF7 in monocytes was a shared feature among MCTD, SLE, and pSS (Fig. 6D). These analyses identified the upstream regulons driving cell-type-specific state transitions toward disease.

### Cell-extrinsic interactions in MCTD, SLE, and pSS

CellChat was used to investigate potential interactions between immune cells in SLE compared to HCs, as well as in pSS compared to HCs. Some signaling pathways activities were upregulated, including IFN-II, CD40, and CD86, uniquely in SLE patients (Fig. 6A). Meanwhile, the IFN-II, CD40, and BTLA (B- and T-lymphocyte attenuator) signaling pathways were uniquely upregulated in pSS patients (Fig. 6B). Interestingly, monocytes also exhibited the properties of target cells (receivers) in the IFN-II signaling pathway across MCTD, SLE, and pSS patients. The CD40 signaling pathway, with CD4^+^ Tm cells as the source cells targeting monocytes, was a shared feature in both SLE and pSS. The upregulated CD86 and BTLA signaling pathways were distinctly characteristic of SLE and pSS, respectively (Fig. 6D).

## Discussion

MCTD is an uncommon connective tissue disease characterized by the presence of anti-U1RNP antibodies. This study aimed to understand the cellular transcriptional changes in MCTD patients. It is the first to create a high-resolution atlas and systematically discuss the cellular heterogeneity in MCTD patients. The scRNA-seq was performed on PBMCs, followed by cell type annotation; DEGs and biological feature analyses; as well as gene regulatory network and cell–cell communication analyses. At this resolution, 15 cell types were identified, and CD8^+^ effector T cells were expanded while γδ T cells were significantly decreased in MCTD patients. “IFN γ response” and “IFN α response” related genes were significantly elevated in MCTD patients, particularly in monocytes. SCENIC analysis provided clues for identifying candidate TFs involved in monocyte dysfunction. Cell communication analysis also offered novel insights into the pathogenesis of MCTD. In summary, this work presents a comprehensive single-cell transcriptome atlas for MCTD, contributing to a more detailed understanding of its pathogenesis and offering new insights into potential diagnostic biomarkers for prospective therapeutic interventions.

MCTD, SLE, and pSS are complex autoimmune diseases. The immune cell dysfunctions and abnormal signaling molecules still require further mechanistic examination and validation. The scRNA-seq analysis of PBMCs from MCTD, SLE, and pSS patients, as well as HCs, allows for the first unbiased, de novo identification of distinct cell types for all three ADs. This study, identifying cell subtype alterations, biological features, gene regulatory networks, and cellular interactions, provides system-level insights based on molecular data (Fig. 6E). These analyses helped clarify the role of shared and distinct immune cell types in the pathogenesis of MCTD, SLE, and pSS, which may guide the discovery for prospective drug targets for these conditions.

In this study, elevated CD8^+^ effector T cells and a decrease in MAIT cells were common in MCTD, SLE, and pSS. CD4^+^ naïve T cells were reduced in both SLE and pSS. The decreased proportions of γδ T cells, B cells, and CD8^+^ naïve T cells were characteristic of MCTD, SLE, and pSS, respectively. These findings align with prior research on SLE and pSS. Some studies have shown that, compared with HCs, the proportion of B cells and CD4^+^ T cells was sharply decreased, while the proportion of CD8^+^ T cells and cytotoxic T cells (CD8^+^ CD28^+^) was prominently higher in SLE patients [38–40]. A reduction in naïve CD4^+^ T cells and an increase in repertoire-restricted GZMH^+^ CD8^+^ T cells have been observed in PBMCs from SLE patients [41]. MAIT cells, which contribute to protection against certain microorganism infections and play an important role in mucosal immunity, have been observed numerically and functionally deficient in cases of SLE [42]. Previous studies have also demonstrated that the peripheral CD4^+^ T cells were decreased in pSS patients. Abnormal proliferation of CD8^+^ T lymphocytes can be detected in the peripheral circulation and specific target tissues from pSS patients [43, 44]. Further understanding of the pathogenic or regulatory effects of activated CD8^+^ T subsets is expected to provide effective treatments for pSS patients in the future [45]. In PBMCs from pSS patients, MAIT cells have been observed as both reduced and functionally immature [46]. In summary, this research established the shared and distinct characteristics of peripheral blood immune cell proportions in MCTD, SLE, and pSS, concurring with previous findings.

There was significant overlap in the biological features of MCTD, SLE, and pSS observed in this study. In the over-representation analysis, the DEGs in MCTD, SLE, and pSS were enriched in identical pathways, mainly the IFN γ response and IFN α response. Type I IFN and IFN γ are pleiotropic cytokines that act as a bridge between innate and adaptive immunity, playing crucial roles in immunity and inflammation regulation [47, 48]. They contribute to ADs by supporting antigen presentation; regulating DC maturation and macrophage activation; as well as inducing the expression of chemokines, cytokines, and inflammatory factors and so on [34, 49]. To date, numerous studies have focused on the pathogenic role of IFNs in ADs. Serum levels of IFN-α/-β/-γ were increased in MCTD patients, and genetic variants of *IFN-α* and *IFN-*γ have shown significant association with the occurrence of MCTD [28, 50]. Decreased DNA methylation in MCTD patients compared with controls has been identified in genes that are transcriptionally responsive to IFN or type I IFN pathways [51]. Until now, there has been no published single-cell transcriptional profiling for MCTD. The results here further confirm the pathogenicity of IFN genes in MCTD at single-cell transcriptome level, and support the potential of IFN genes as candidates for MCTD susceptibility. Several lines of evidence have emphasized the roles for type I and type II IFN in the pathogenesis of SLE and pSS [30, 32–34, 52–54]. The findings from this study align with prior research on SLE and pSS. Anifrolumab, a human monoclonal antibody targeting type I IFN receptor subunit 1, is currently authorized for moderate-to-severe SLE [55, 56]. IFN α kinoid (IFN-K) has been shown to induce neutralizing anti-IFN-α2b antibodies and significantly reduce the IFN signature with acceptable safety in active adult SLE [57]. Taken together, the results presented here strongly support the notion that IFN-related genes could serve as potential therapeutic targets for MCTD, SLE, and pSS.

Monocytes are pivotal in promoting and regulating inflammation in SLE. The scRNA-seq datasets revealed disease activity-dependent expansion of SLE-specific monocyte subsets and supported the IFN signature for classic monocytes [58]. There are three biomarkers (IFI30, BLVRA, and RIN2) that are involved in IFN-related signaling pathways and act as SLE-associated biomarkers of monocytes [59]. Monocytes are hyperresponsive to stimulation of the IFN related genes, and play a critical role in the pathogenesis of pSS [60, 61]. An increased proportion of CD226 on CD14^+^ monocytes was associated with the clinical manifestations, disease activity, and prognosis of pSS patients. CD226^+^ CD14^+^ monocytes may present a potential target and a biomarker for the prognosis and therapy of pSS patients [62]. Based on the study results, we inferred that monocytes may play a pivotal role in the pathogenesis of MCTD, which requires further validation.

The transcriptional regulatory analysis identified several TFs that are highly related to the pathogenesis of MCTD, SLE, and pSS. Increased STAT1 and IRF7 activities in monocytes were shared across the MCTD, SLE, and pSS. Combined with the regulatory network described results described in previous results, these shared TF target genes related to IFN response. The results suggest that MCTD shares the same IFN signature as SLE and pSS. Numerous studies have demonstrated that the signal transducer and activator of transcription (STAT) families and IFN regulatory factor (IRF) play vital roles in IFN response [63]. The Janus kinase/signal transduction and activator of transcription (JAK/STAT) signaling pathway drives diverse immune regulatory processes, including cell proliferation, survival, inflammation, and immune tolerance. Aberrant JAK/STAT transduction jeopardizes immune balance and contributes to the ADs development [64–66]. Tofacitinib is a selective inhibitor of JAK1 and/or JAK3. It can target the synovial JAK/STAT signaling pathway in RA, reducing the expression of matrix metalloproteinases (MMPs) and IFN-regulated genes in synovial cells [67]. A previous study confirmed that tofacitinib partially improved arthritis and rash in patients with SLE [68]. Some studies also suggested that tofacitinib could be used as an anti-inflammatory and antifibrotic agent in pSS patients, showing potential for the treatment of pSS-associated interstitial lung disease (ILD) [69, 70]. The results here indicate that tofacitinib might also be an effective drug for MCTD patients. As JAK substrates and type I/II cytokine receptors downstream, STATs have been studied as attractive targets for the treatment of inflammation, autoimmunity, and malignancies. Unfortunately, most STAT inhibitors are still in preclinical development, and few have been approved for clinical application due to issues with bioavailability and selectivity. A large number of clinical trials are currently underway, targeting STATs both as monotherapy or combination therapy, providing an attractive drug target [64]. IRFs can induce the expression of IFN-stimulated genes, either dependent or independent of JAK-STAT signaling. IRF7 has been found to be pivotal in SLE and pSS [71, 72]. The results of this study further confirm the role of IRF7 in the pathogenesis of SLE and pSS, in addition to MCTD.

The study aimed to investigate cellular communication within PBMCs through ligand-receptor interactions, which revealed an increased IFN-II signaling network, with monocytes as the recipient cells in MCTD, SLE, and pSS. The findings were consistent with the DEGs over-representation and transcriptional regulatory analyses. The CD40 signaling pathway network was most enriched from CD4^+^ memory T cells to monocytes in both SLE and pSS. The CD40 receptor and its ligand, CD40L, are among the most critical molecular pairs of the stimulatory immune checkpoints. Due to its essential role in immune activation, CD40/CD40L interaction has been regarded as an attractive immunotherapy target [73]. Both CD4^+^ and CD8^+^ cells from SLE patients have shown upregulated CD40L expression [74, 75]. A number of anti-CD40L and anti-CD40 drugs (antibodies), with a variety of biological effects, are in clinical trials for ADs treatment. Iscalimab, a CD40-targeted antagonist, is undergoing multiple clinical trials in SLE and Sjögren’s syndrome [76]. Dapirolizumab, a CD40L antagonistic monoclonal antibody (mAb), showed potential efficacy in a phase 1 trial with SLE patients, particularly with high disease activity [77]. The results here confirm that CD40 and CD40L may be potent drug targets not only in SLE but also in pSS. CD70 and CD27 constitute a ligand-receptor pair within the TNF ligand and receptor family, which plays a major role in T-cell co-stimulation. CD70 has been shown to be highly abundant in CD4^+^ T cells from RA [78] and SLE patients [79]. Increased CD27/CD70 signaling has also been reported in myasthenia gravis (MG) patients [80]. The results of this study showed that the CD70 signaling network was highly enriched in MCTD. Anti-CD70 mAb have been shown to reduce T-cell-dependent colitis [81] and ameliorate bone and cartilage destruction in collagen-induced arthritis [82], suggesting that CD70 may be a viable target for immune intervention. The CD80/CD86-CD28 costimulatory signals play an important role in the SLE occurrence and development. Excessive expression of CD80 and CD86 molecules has been observed on freshly isolated B cells in SLE patients [83]. The CD86 signaling pathway (CD86-CD28) was observed to be enriched in SLE, which is consistent with the previous reports. An anti-CD80 mAb has been employed to inhibit immune response and attenuate severity of a murine lupus model [84]. A mAb against human CD86(1D1) has been developed which could prevent the development of chronic graft-versus-host disease (cGVHD)-induced lupus [85]. BTLA, which belongs to the CD28 superfamily, is an immuno-inhibitory receptor with the ability to suppress lymphocyte activation. The BTLA ligand, herpesvirus entry mediator (HVEM), is a member of the tumor necrosis factor receptor (TNFR) superfamily. Increased expression of BTLA has been reported in T lymphocytes from RA patients [86]. Additionally, PBMCs from RA patients showed high surface expression of HVEM [87]. This study revealed that BTLA signaling network was enriched in pSS. The BTLA-HVEM complex may be another immune checkpoint that could be targeted for the ADs treatment and warrants further investigation.

Additionally, this study has several limitations. First, the sample size is limited. The etiology of MCTD is complex, and significant individual differences exist among MCTD patients, which warrant further investigation in large-scale studies. Second, the analysis results are speculative, and further experimental research, including immunoblotting, is needed to confirm these findings. Finally, the potential relationship between monocytes and disease phenotypes requires further validation, which will be the focus of our future work.

In conclusion, this study conducted scRNA-seq analyses of diverse autoimmune diseases (MCTD, SLE, and pSS) using our transcriptome sequencing and GEO datasets. The results demonstrated shared and distinct alterations in cell subtypes, abnormal biological features, changes in TF activities, and cell communication networks across the three diseases. This provides a system-level understanding based on molecular data. These findings help clarify the role of immune cells in the pathogenesis of MCTD, SLE, and pSS, and highlight potential targets for treating these conditions.

## Supplementary Information

Additional file 1.

## Abbreviations

MCTD: Mixed connective tissue disease
SLE: Systemic lupus erythematosus
pSS: Primary Sjögren’s syndrome
ADs: Autoimmune diseases
scRNA-seq: Single-cell RNA-sequencing
PBMCs: Peripheral blood mononuclear cells
HVGs: Highly variable genes
PCA: Principal component analysis
UMAP: Uniform manifold approximation and projection
AUC: Average regulon activity
GEO: Gene Expression Omnibus
