# Supplementary material for: Shared and distinct peripheral blood immune cell landscape in MCTD, SLE, and pSS
Source: Cell Biosci. 2025 Apr 10;15:42. doi: 10.1186/s13578-025-01374-1 (PMC11983850; doi:10.1186/s13578-025-01374-1)
Supplement: Supplementary file 1 — Additional file 1. [file 13578_2025_1374_MOESM1_ESM.docx]

**Shared and distinct peripheral blood immune cell landscape in MCTD, SLE, and pSS**

Yanling Cui ^1†^, Huina Zhang ^1†^, Yaxuan Deng ^1,2†^, Orion Fan ^1,2^, Junbang Wang ^1^, Zhonggang Xing ^1,2^, Jianping Tang ^3^^*^, Wenmin Zhu ^1*^, Bangdong Gong ^3*^, and Yi Eve Sun ^1,2*^

**Supplementary figure 1**


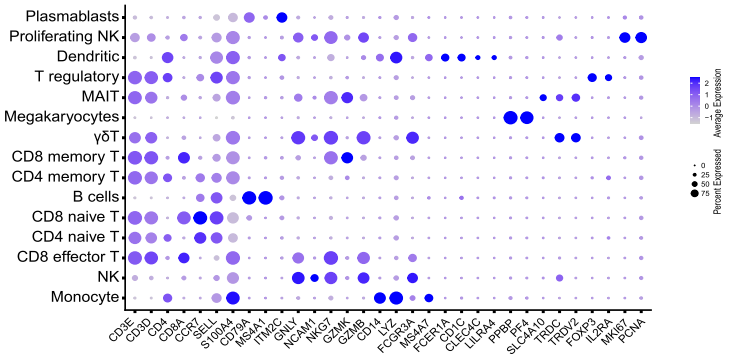


Dot plot showing the classical marker gene expression used for cluster cell type annotation. Darker blue colors indicate elevated gene expression levels, and dot sizes represent the percentage of cells.

**Supplementary figure 2**


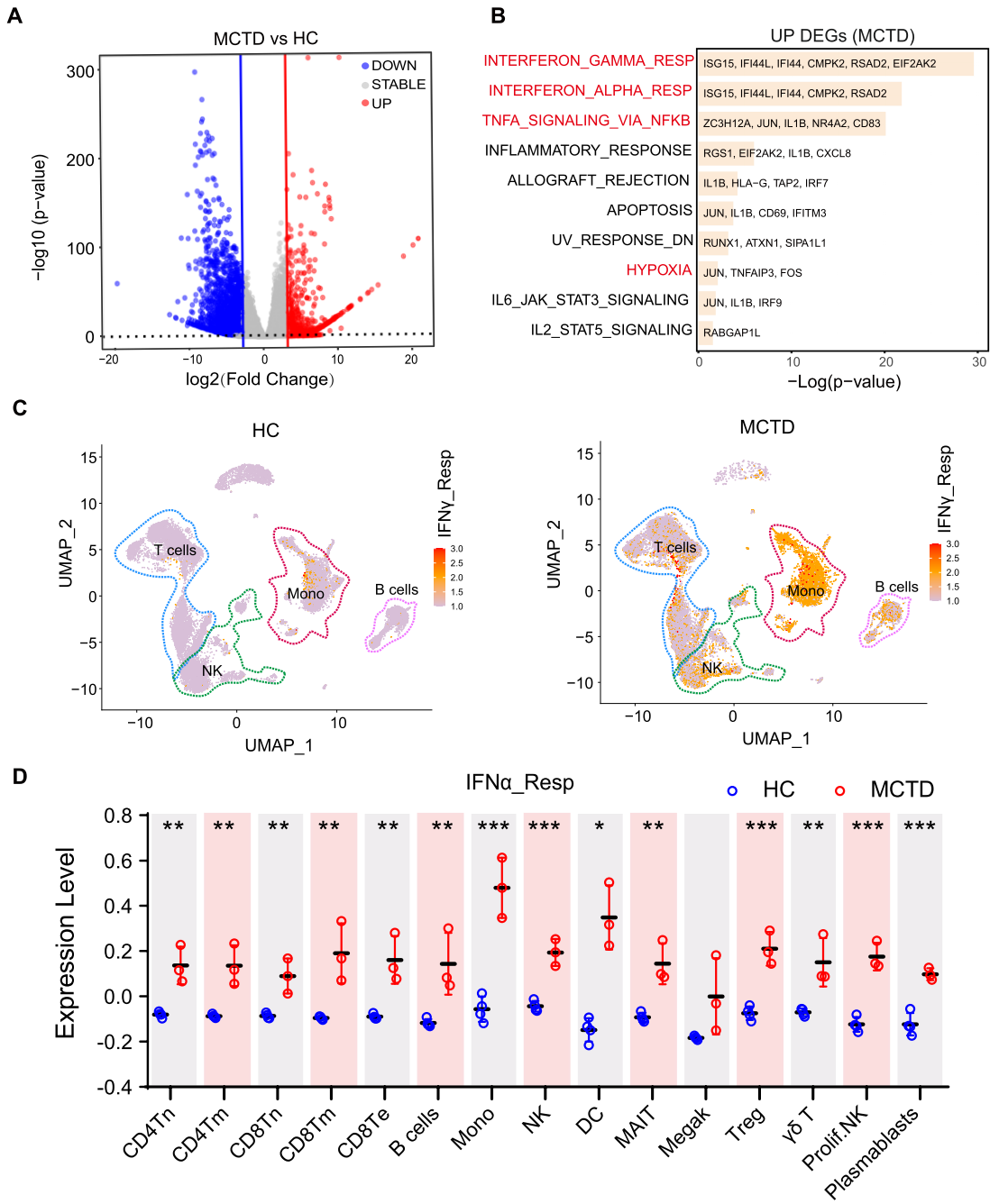


(A) Volcano plot of DEGs in RNA-seq between MCTD and HCs. Red dots indicate upregulated genes and blue dots indicate downregulated genes.

(B) Functional enrichment analysis of upregulated DEGs in MCTD.

(C) UMAP visualization colored by the “IFN γ response” expression scores in MCTD and HCs.

(D) Scatter dot plot depicting the “IFN α response” expression score of each cell type in MCTD and HCs.

**Supplementary figure 3**


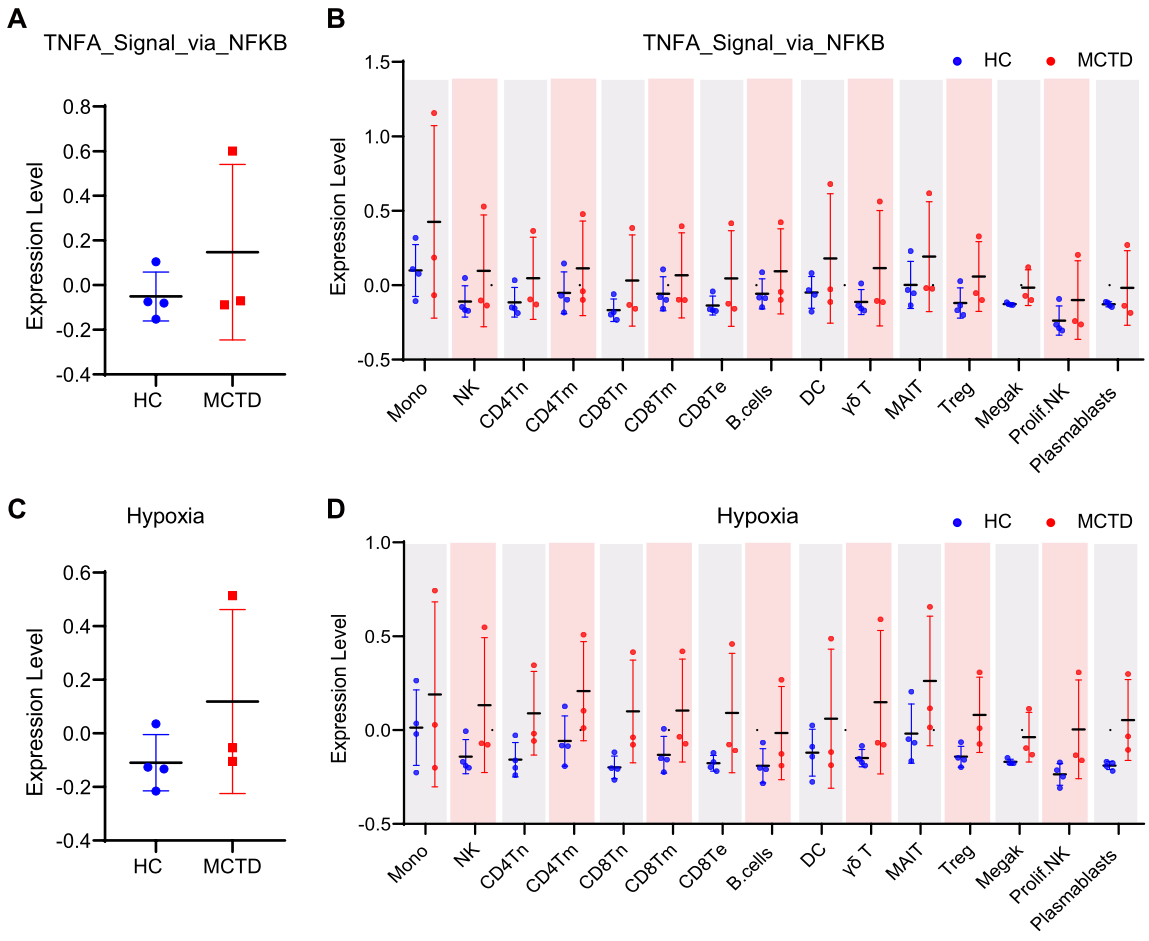


Scatter dot plot depicting overall (A) “TNFα signaling via NF-κB” expression score and (C) “Hypoxia” expression score between MCTD and HCs.

Scatter dot plot depicting (B) “TNFα signaling via NF-κB” expression score and (D) “Hypoxia” expression score of each cell type between MCTD and HCs.

**Supplementary figure 4**


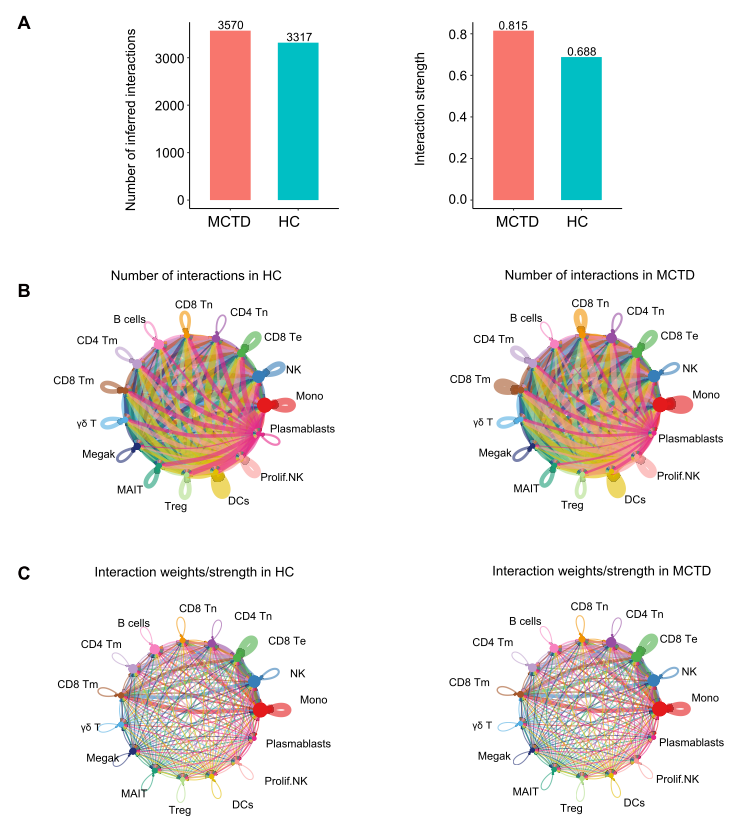


(A) Overall number and intensity of ligand-receptor interactions among cell types.

Circle plots showing (B) the numbers of interactions and (C) the strengths of interactions among different cell types in MCTD and HCs.

**Supplementary figure 5**


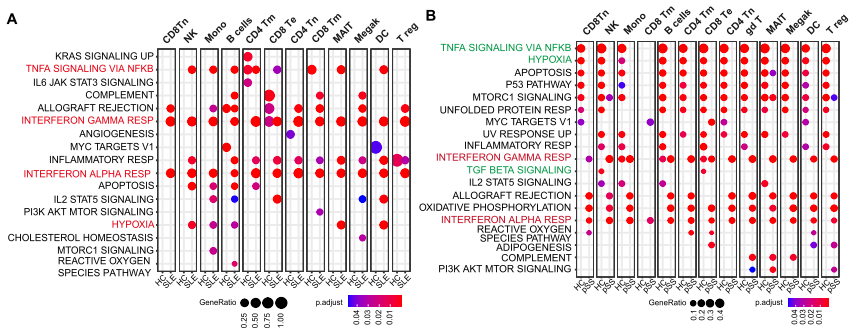


Over-representation analysis of HALLMARK gene sets among each cell types demonstrated common changes in gene sets representing altered immunological states between (A) SLE and HCs, (B) pSS and HCs.

**Supplementary figure6**


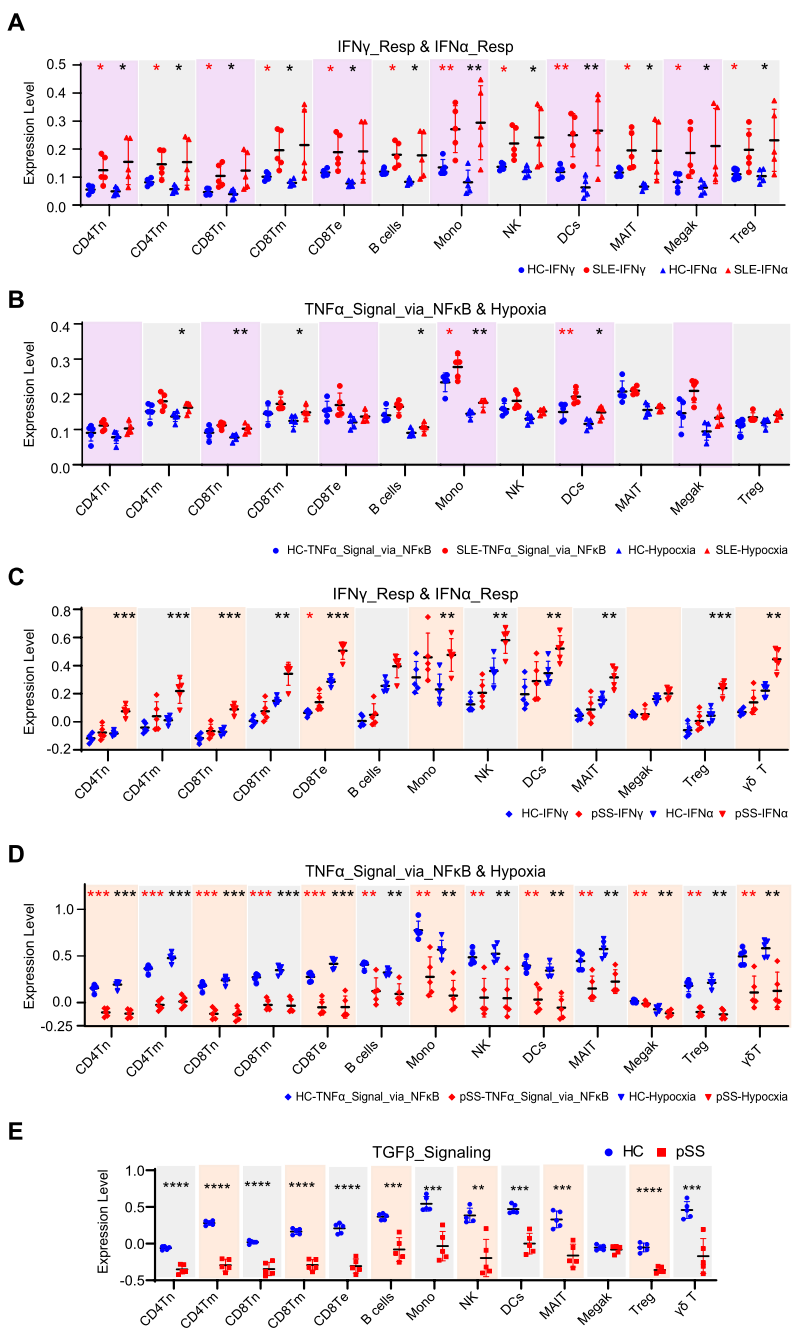


Scatter dot plot depicting (A) “IFN γ response” and “IFN α response” expression scores, (B) “TNFα signaling via NF-κB” and “Hypoxia” expression scores in each cell type between SLE and HCs. (C) “IFN γ response” and “IFN α response” expression scores, (D) “TNFα signaling via NF-κB” and “Hypoxia” expression scores, and (E) “TGFβ signaling” expression score in each cell type between pSS and HCs.

**Supplementary Table 1.**

**Baseline clinical characteristics of MCTD patients and HCs for single-cell sequencing.**

|  | Patient1 | Patient2 | Patient3 | HC1 | HC2 | HC3 | HC4 |
| --- | --- | --- | --- | --- | --- | --- | --- |
| Age (year) | 29 | 24 | 51 | 26 | 30 | 56 | 47 |
| Gender | F | F | F | M | M | F | F |
| Race | Han | Han | Han | Han | Han | Han | Han |
| Group | MCTD | MCTD | MCTD | HC | HC | HC | HC |
| Disease duration (month) | 6 | 36 | 6 | **/** | **/** | **/** | **/** |
| Raynaud phenomenon | YES | YES | YES | **/** | **/** | **/** | **/** |
| Swollen hands | YES | YES | YES | **/** | **/** | **/** | **/** |
| Arthralgia | NO | YES | YES | **/** | **/** | **/** | **/** |
| Myalgia | YES | NO | NO | **/** | **/** | **/** | **/** |
| Rash | NO | NO | YES | **/** | **/** | **/** | **/** |
| Fever | YES | NO | NO | **/** | **/** | **/** | **/** |
| Fatigue | YES | YES | YES | **/** | **/** | **/** | **/** |
| Interstitial lung disease | YES | NO | NO | **/** | **/** | **/** | **/** |
| Pulmonary hypertension | YES | NO | NO | **/** | **/** | **/** | **/** |
| Hb (g/L) | 91↓ | 120 | 135 | **/** | **/** | **/** | **/** |
| WBC (×10^9^/L) | 2.82↓ | 3.46↓ | 5.36 | **/** | **/** | **/** | **/** |
| PLT (×10^9^/L) | 193 | 206 | 245 | **/** | **/** | **/** | **/** |
| CRP (mg/L) | <0.5 | 2.4 | 5.14 | **/** | **/** | **/** | **/** |
| ESR (mm/h) | 40↑ | 30↑ | 29↑ | **/** | **/** | **/** | **/** |
| ANA≥1:100 | YES | YES | YES | **/** | **/** | **/** | **/** |
| U1RNP positive | YES | YES | YES | **/** | **/** | **/** | **/** |
| Serum IgG (g/L) | 17.5↑ | 22.9↑ | 17.4↑ | **/** | **/** | **/** | **/** |
| Serum IgA (g/L) | 2.09 | 3.42 | 3.44 | **/** | **/** | **/** | **/** |
| Serum IgM (g/L) | 0.5 | 1.13 | 0.95 | **/** | **/** | **/** | **/** |
| Serum C3 (g/L) | 0.72↓ | 0.9 | 0.98 | **/** | **/** | **/** | **/** |
| Serum C4 (g/L) | 0.11 | 0.24 | 0.29 | **/** | **/** | **/** | **/** |
| RF (IU/mL) | <20 | 174↑ | <20 | **/** | **/** | **/** | **/** |
| Treatment duration  before enrollment (month) | 0 | 0 | 0 | **/** | **/** | **/** | **/** |
| Previous medication | **/** | **/** | **/** | **/** | **/** | **/** | **/** |

ANA: antinuclear antibody, U1RNP: U1 ribonucleoprotein.

**Supplementary Table 2.**

**Clinical characteristics of MCTD patients for qPCR.**

|  | MCTD1 | MCTD2 | MCTD3 | MCTD4 | MCTD5 | MCTD6 | MCTD7 |
| --- | --- | --- | --- | --- | --- | --- | --- |
| Age (year) | 32 | 34 | 49 | 35 | 53 | 37 | 46 |
| Gender | F | F | F | F | F | F | F |
| Race | Han | Han | Han | Han | Han | Han | Han |
| Disease duration (month) | 24 | 1 | 84 | 24 | 36 | 60 | 120 |
| Raynaud phenomenon | YES | YES | YES | YES | YES | YES | YES |
| Swollen hands | YES | YES | YES | YES | YES | YES | YES |
| Arthralgia | NO | NO | NO | NO | YES | YES | NO |
| Myalgia | NO | NO | NO | NO | YES | YES | NO |
| Rash | YES | YES | NO | YES | NO | NO | YES |
| Fever | NO | YES | NO | NO | NO | NO | NO |
| Fatigue | NO | YES | NO | YES | NO | NO | YES |
| Interstitial lung disease | NO | NO | YES | YES | YES | NO | NO |
| Pulmonary hypertension | NO | NO | NO | NO | NO | NO | YES |
| Hb (g/L) | 129 | 147 | 94↓ | 112↓ | 119 | 123 | 129 |
| WBC (×10^9^/L) | 8.02 | 2.5↓ | 3.59 | 3.12↓ | 5.64 | 5.39 | 3.77 |
| PLT (×10^9^/L) | 255 | 162 | 70↓ | 268 | 179 | 263 | 156 |
| CRP (mg/L) | <0.5 | 8 | 2.28 | 1.4 | 0.62 | 0.71 | <0.5 |
| ESR (mm/h) | 35↑ | 4 | 45↑ | 47↑ | 11 | 9 | 6 |
| ANA≥1:100 | YES | YES | YES | YES | YES | YES | YES |
| U1RNP positive | YES | YES | YES | YES | YES | YES | YES |
| Serum IgG (g/L) | 19.1↑ | 12.7 | 17.3↑ | 20.6↑ | 9.98 | 21.6↑ | 14.4 |
| Serum IgA (g/L) | 4.94↑ | 2.21 | 4.45 | 4.88↑ | 1.83 | 3.71 | 2.13 |
| Serum IgM (g/L) | 1.93 | 0.63 | 3.11↑ | 1.19 | 1.45 | 0.69 | 0.36↓ |
| Serum C3 (g/L) | 1.12 | 0.99 | 0.79 | 0.91 | 0.69↓ | 0.55↓ | 0.72↓ |
| Serum C4 (g/L) | 0.24 | 0.33 | 0.14↓ | 0.21 | 0.07↓ | 0.08↓ | 0.17 |
| RF (IU/mL) | <20 | <20 | 23.2↑ | 95.5↑ | 48↑ | <20 | <20 |
| Treatment duration  before enrollment (month) | 0 | 0 | 0 | 0 | 0 | 0 | 0 |
| Previous medication | **/** | **/** | **/** | **/** | **/** | **/** | **/** |

ANA: antinuclear antibody, U1RNP: U1 ribonucleoprotein.

**Supplementary Table 3.**

**Clinical characteristics of SLE patients for qPCR.**

|  | SLE1 | SLE2 | SLE3 | SLE4 | SLE5 | SLE6 | SLE7 | SLE8 | SLE9 | SLE10 |
| --- | --- | --- | --- | --- | --- | --- | --- | --- | --- | --- |
| Age (year) | 48 | 64 | 63 | 55 | 42 | 42 | 60 | 19 | 38 | 42 |
| Gender | F | F | F | F | F | F | F | F | F | F |
| Race | Han | Han | Han | Han | Han | Han | Han | Han | Han | Han |
| Disease duration (month) | 180 | 264 | 240 | 120 | 10 | 108 | 24 | 12 | 24 | 384 |
| ANA≥1:100 | (+) | (+) | (+) | (+) | (+) | (+) | (+) | (+) | (+) | (+) |
| Anti-Sm | (-) | (-) | (-) | (-) | (+) | (-) | (-) | (+) | (+) | (-) |
| Anti-dsDNA | 344↑ | 31 | 4.9↑ | 301↑ | 702↑ | 323↑ | 837↑ | <10 | 245↑ | 369↑ |
| Articular | YES | YES | YES | YES | NO | NO | NO | NO | NO | NO |
| Muscular | NO | NO | NO | NO | NO | YES | NO | YES | NO | NO |
| Rash | NO | YES | YES | NO | NO | YES | YES | YES | NO | YES |
| Fever | YES | YES | YES | NO | YES | NO | NO | YES | YES | NO |
| Renal | NO | NO | NO | NO | NO | NO | NO | NO | NO | YES |
| Hematological | YES | YES | NO | NO | YES | YES | YES | NO | YES | YES |
| nervous involvement | YES | NO | NO | YES | NO | NO | NO | YES | NO | NO |
| Hb (g/L) | 123 | 119 | 130 | 131 | 88↓ | 94↓ | 117 | 140 | 121↓ | 99↓ |
| WBC (×10^9^/L) | 4.58 | 3.25↓ | 5.4 | 5.17 | 3.16↓ | 1.93↓ | 3.1↓ | 3.79 | 1.36↓ | 10.2↑ |
| PLT (× 10^9^/L) | 57↓ | 176 | 186 | 167 | 128 | 142 | 43↓ | 259 | 140 | 47↓ |
| CRP (mg/L) | 5.1 | <0.5 | 2.01 | 2 | 5 | 2.7 | 0.8 | 22.4↑ | 32.9↑ | 45.7↑ |
| ESR (mm/h) | 5 | 19 | 23 | 12 | 55↑ | 55↑ | 68↑ | 40↑ | 66↑ | 10 |
| RF(IU/ml) | <20 | <20 | <20 | <20 | 28.3↑ | <20 | 27↑ | 66.7↑ | <20 | <20 |
| β2-GP1 | (-) | (-) | (-) | (-) | (-) | (-) | (-) | (-) | (-) | (-) |
| ACL | 27.27 | 23.52 | 23.9 | 13.6 | 16 | 44.4↑ | 2.4 | 12 | 26.82 | 12.36 |
| Serum IgG (g/L) | 16.8↑ | 12.4 | 12.8 | 23.2↑ | 28↑ | 19.9↑ | 37.9↑ | 10.2 | 24.5↑ | 12.5 |
| Serum IgA (g/L) | 2.34 | 2.92 | 6.31↑ | 5.4↑ | 6.28↑ | 6.24↑ | 3.29 | 2.48 | 4.68↑ | 4.57↑ |
| Serum IgM (g/L) | 1.21 | 0.61 | 0.25↓ | 0.89 | 0.46 | 1.6 | 0.63 | 1.23 | 0.62 | 1.01 |
| Serum C3 (g/L) | 0.43↓ | 0.56↓ | 0.74↓ | 0.92 | 0.48↓ | 0.59↓ | 0.75↓ | 0.93 | 0.62↓ | 0.45↓ |
| Serum C4 (g/L) | 0.09↓ | 0.14↓ | 0.19 | 0.18 | 0.07↓ | 0.11↓ | 0.09↓ | 0.14↓ | 0.18 | 0.12↓ |
| 24h urinary protein | 0.04 | 0.06 | 0.05 | 0.1 | 0.28↑ | 0.25↑ | 0.05 | 0.18↑ | 0.03 | 0.08 |
| SLEDAI | 12 | 8 | 6 | 10 | 11 | 9 | 5 | 14 | 10 | 13 |
| Treatment duration  before enrollment (month) | 168 | 264 | 240 | 0 | 0 | 96 | 0 | 0 | 0 | 120 |
| Previous medication | GCs, HCQ,  CsA | GCs, HCQ,  CsA | GCs, AZA,  LEF | **/** | GCs,  Tacrolimus | GCs, HCQ,  Tacrolimus | GCs, HCQ, CsA | GCs, HCQ,  MMF, | GCs, HCQ | GCs, MMF, Tacrolimus |

ANA: antinuclear antibody, Anti-Sm: [Anti-Smith, Anti-dsDNA: Anti-double stranded DNA,](https://medilinx.com.ph/product/anti-smith-anti-sm/) GCs: Glucocorticoids, HCQ: Hydroxychloroquine, CsA: Cyclosporin A, AZA: Azathioprine, LEF: Leflunomide, MMF: Mycophenolate mofetil.

**Supplementary Table 4.**

**Clinical characteristics of pSS patients for qPCR.**

|  | pSS1 | pSS2 | pSS3 | pSS4 | pSS5 | pSS6 | pSS7 | pSS8 |
| --- | --- | --- | --- | --- | --- | --- | --- | --- |
| Age (year) | 62 | 42 | 63 | 56 | 45 | 58 | 60 | 61 |
| Gender | F | F | F | F | F | F | F | F |
| Race | Han | Han | Han | Han | Han | Han | Han | Han |
| Disease duration (month) | 6 | 48 | 120 | 60 | 120 | 48 | 120 | 60 |
| Focus score of ≥1 | (-) | (+) | (+) | (+) | (+) | (+) | (+) | (+) |
| ANA≥1:100 | (+) | (-) | (+) | (-) | (+) | (+) | （＋） | (+) |
| Anti-SSA/Ro | (+) | (+) | (+) | (-) | (+) | (+) | (-) | (+) |
| Anti-SSB/La | (-) | (+) | (-) | (-) | (+) | (+) | (-) | (+) |
| Articular | NO | YES | NO | YES | YES | YES | NO | NO |
| Muscular | NO | YES | NO | NO | YES | NO | NO | NO |
| Rash | NO | NO | NO | NO | NO | NO | NO | YES |
| Hematological | NO | NO | YES | NO | YES | YES | YES | YES |
| Renal | NO | NO | NO | NO | NO | NO | NO | NO |
| ILD | NO | NO | YES | NO | NO | YES | NO | YES |
| nervous involvement | NO | YES | NO | NO | NO | NO | NO | YES |
| Schirmer’s test | (+) | (+) | (+) | (+) | (+) | (+) | (+) | (+) |
| Hb (g/L) | 146 | 116 | 92↓ | 120 | 132 | 122 | 114↓ | 117 |
| WBC (×10^9^/L) | 6.94 | 8.98 | 5 | 6.41 | 2.82↓ | 4.33 | 3.31↓ | 4.25 |
| PLT (×10^9^/L) | 328 | 180 | 180 | 251 | 244 | 109↓ | 220 | 87↓ |
| CRP (mg/L) | <0.5 | 2.6 | <0.5 | <0.5 | 0.68 | 0.55 | <0.5 | 3.08 |
| ESR (mm/h) | 34 | 19 | 20 | 9 | 76↑ | 35 | 2 | 37 |
| RF (IU/ml) | <20 | <20 | <20 | <20 | 61↑ | 20 | <20 | <20 |
| IgG(g/l) | 14.3 | 10.4 | 11.8 | 11.2 | 22.2↑ | 16.6↑ | 9.14 | 20.8↑ |
| Serum IgA (g/L) | 2.23 | 1.76 | 0.66↓ | 0.8↓ | 8.43↑ | 3.2 | 1.03 | 3.32 |
| Serum IgM (g/L) | 0.85 | 1.21 | 0.63 | 0.83 | 0.85 | 2.98 | 0.16↓ | 1.59 |
| Serum C3 (g/L) | 0.89 | 1.27 | 0.62↓ | 0.88 | 1.11 | 0.89 | 0.63↓ | 0.85 |
| Serum C4 (g/L) | 0.21 | 0.31 | 0.14↓ | 0.19 | 0.22 | 0.2 | 0.19 | 0.21 |
| ESSDAI | 2 | 5 | 5 | 2 | 10 | 3 | 4 | 17 |
| Treatment duration  before enrollment (month) | 0 | 24 | 120 | 24 | 96 | 48 | 84 | 24 |
| Previous medication | HCQ | HCQ | GCs,  HCQ,  CYC | GCs,  HCQ,  CYC | CsA | GCs,  HCQ,  CsA | **/** | GCs |

ANA: antinuclear antibody, Anti-SSA/Ro: anti–Sjögren's syndrome related antigen A, Anti-SSB/La: anti–Sjögren syndrome type B antigen GCs: Glucocorticoids, HCQ: Hydroxychloroquine, CsA: Cyclosporin A, CYC: Cyclophosphamide.

**Supplementary Table 5.**

**Primer sequences used in this study**

| Target gene | Forward primer (5'--3') | Reverse primer (5'--3') |
| --- | --- | --- |
| h-GAPDH | GGGAAACTGTGGCGTGAT | GAGTGGGTGTCGCTGTTGA |
| h-STAT1 | ATCAGGCTCAGTCGGGGAATA | TGGTCTCGTGTTCTCTGTTCT |
| h-IRF7 | CCCACGCTATACCATCTACCT | GATGTCGTCATAGAGGCTGTTG |
